# Structural and Virus Regulatory Insights Into Avian N6-Methyladenosine (m6A) Machinery

**DOI:** 10.3389/fcell.2020.00543

**Published:** 2020-07-15

**Authors:** Mahmoud Bayoumi, Mohammed A. Rohaim, Muhammad Munir

**Affiliations:** Division of Biomedical and Life Sciences, Lancaster University, Lancaster, United Kingdom

**Keywords:** chicken, epigenetics, evolution, fat mass and obesity-associated, structural insights, avian N6-methyladenosine, methyltransferase like-3, YTHDC1

## Abstract

The addition of a methyl group to the N6 position of adenosine (m6A) is the most common posttranscriptional RNA modification, and it regulates most steps of RNA metabolism including splicing, stability, translation, nuclear-export, and RNA structures. Besides cellular RNA, m6A modifications have also been detected on viral RNA. A range of recent studies have demonstrated the crucial roles of m6A in the virus–host interactions; however, m6A cellular machineries are only characterized in limited mammalian species. Herein, we aim to present comprehensive evolutionary insights into major m6A writers, erasers, and readers and draw a comparative structural analysis between avian and mammalian m6A-associated machineries. The comparative collinearity on the chromosomal scale revealed that the majority of m6A-related genes were found less syntenic even among avian species. Genetic analysis of avian m6A erasers revealed a distinct phylogenetic clustering compared to mammalian orthologs and shared a weak percent (55%) identity with mammalian species with low identity percentage (55%). The overall comparative three-dimensional (3D) structure analyses among different mammalian species were maintained through synonymous structural mutations. Unlike erasers, the putative 3D structures in the active sites as for the aromatic cage in YTH-domain of YTHDC1 and two pivotal loops in MTD-domains in METTL3 exhibited structural alterations in chicken. In conjunction with *in silico* investigations, influenza viruses significantly downregulated gene the transcription of m6A writers and erasers, whereas m6A readers were moderately regulated in chicken fibroblasts. In light of these findings, future detailed biochemical and crystallographic studies are warranted to define the roles of m6A machinery in regulating both viral and cellular RNA metabolism in avian species.

## Introduction

The cellular RNA plays an integral role in the cell life cycle. Posttranscriptional modifications include capping, splicing, and polyadenylation. More than 100 different chemical modifications have been described in various forms of RNAs: tRNA, rRNA, lncRNA, and mRNA. These modifications, collectively referred to as epitranscriptome, display an extensive landscape and affect a variety of biological processes (Lewis et al., 2017; Meyer and Jaffrey, 2017; Roundtree et al., 2017; Huang et al., 2020). The chemical modifications integrated in the mRNA of eukaryotes are attributed to different canonical bases: adenosine (m^1^A; Li et al., 2017; Safra et al., 2017) and cytosine (m^5^C; Motorin et al., 2009). Non-canonical bases accept modifications as well: pseudouridine (Carlile et al., 2014) and inosine (Levanon et al., 2004). However, the most prominent and enriched in RNA modifications is methylation at N6 position of adenosine (m6A; Dominissini et al., 2012; Boccaletto et al., 2018).

Methylated adenosine was firstly discovered a few decades ago in hepatoma cells (Desrosiers et al., 1974) and encountered in a wide range of species including human (Dominissini et al., 2012; Meyer and Jaffrey, 2017; Wang et al., 2017), mouse (Mouse Genome Sequencing Consortium et al., 2002; Dominissini et al., 2012; Jain et al., 2018), plant (Martínez-Pérez et al., 2017; Yue et al., 2019), yeast (Agarwala et al., 2012), and bacteria (Deng et al., 2015). Intriguingly, m6A marks are also encoded in wide arrays of viral transcripts of both DNA and RNA viruses (Krug et al., 1976; Narayan et al., 1987; Courtney et al., 2017; Kennedy et al., 2017; Tan and Gao, 2018; Dang et al., 2019; Wu et al., 2019). The m6A has recently been attributed to RNA metabolism pathways including splicing, stability, translation, and secondary structure (Niu et al., 2013; Li and Mason, 2014; Meyer and Jaffrey, 2014). Additionally, m6A marks are involved in normal homeostasis, embryo development, and fertility, suggesting their pivotal regulatory roles (Zheng et al., 2013; Li et al., 2014) in cellular life cycles.

The m6A modification is co-transcriptionally and posttranscriptionally added mainly to the nuclear pre-mRNAs by methyltransferase complex, consisting principally of methyltransferase like-3 (METTL3) and two cofactors, methyltransferase like-14 (METTL14), and Wilms’ tumor 1-associating protein (WTAP), collectively known as m6A “writers” (Niu et al., 2013; Śledź and Jinek, 2016; Wang et al., 2016a, b; Huang and Yin, 2018). Conceivably, upon entry of the mRNAs into the cytoplasm, the methylated RNA is recognized and read by m6A “readers,” proteins containing YT521-B homology (YTH) domain entitled YTHDF1, YTHDF2, and YTHDF3, which are thought to mediate many of the phenotypic effects exerted by m6A (Wang et al., 2015; Shi et al., 2017; Liao et al., 2018). Recently, two additional readers were detected named YTH domain containing proteins: YTHDC1 (nuclear reader) and YTHDC-2 (Xu et al., 2014b; Xiao et al., 2016; Jain et al., 2018). Additionally, two main RNA demethylases “erasers” belonging to Fe(II)/2-oxoglutarate (2OG) dioxygenase superfamily, named AlkB homology 5 (ALKBH5), and Fat mass and obesity-associated (FTO) protein, have been described which can reversibly remove the m6A marks from the RNA (Han et al., 2010; Aik et al., 2014; Xu et al., 2014a).

The m6A methylome and m6A cellular machineries are not genetically or functionally characterized in several organisms species including avian species. However, the availability of next-generation sequencing (NGS) data from an increasing number of species provides exceptional sources in decoding the information contained within these mega data. Comparative analysis of genome sequences from different species that have varying evolutionary distances is deemed for recognizing the functional genes (tend to evolve slowly), non-functional genes (evolve more rapidly), and evolutionary related gene sequences. These datasets additionally provide fundamental knowledge on the host–pathogen interactions (Frazer et al., 2003). Specifically, among avian species, chicken are considered a model for non-mammalian amniote, successor of the dinosaurs, that have joined the list of fully sequenced animal species (International Chicken Genome Sequencing Consortium, 2004). Furthermore, comparison of avian species with a wide range of eukaryotic mammals has empowered the delineation of crucial vertebrate genes. In this study, we comprehensively address the structural and evolutionary changes in m6A cellular machinery proteins of some avian species and compare them with various mammalian species including reptiles, amphibians, and fish. The comparison includes genetic diversity, phylodynamics, evolutionary distances, and the predicted m6A-related proteins with human protein orthologs. These analyses of m6A in avian species will stimulate functional annotation of avian m6A machinery in the foreseeable future, which could contribute to animal genetic improvement and pathogen interference through epitranscriptome manipulations.

## Results

### Loss of Conserved Synteny Among Majority of m6A-Related Orthologs

**FIGURE 1:**
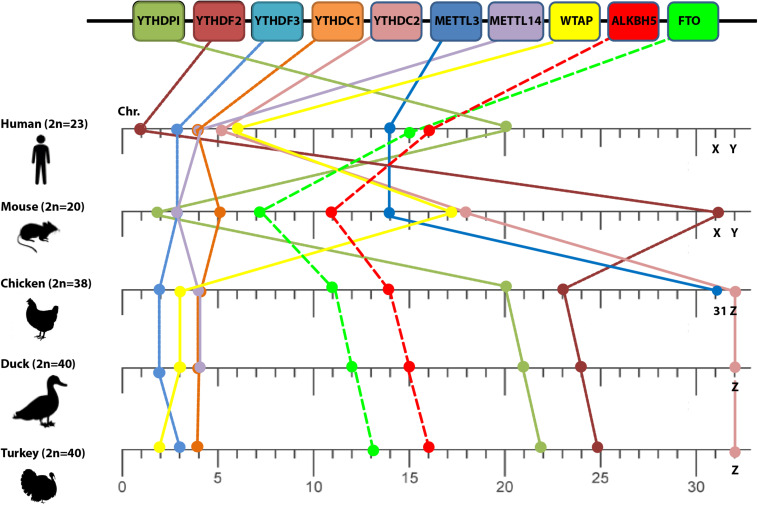
Loss of conserved synteny among different m6A orthologs. Chromosome numbers are indicated on the lower scale bar by numbers (except *X*, *Y*, and *Z* sex chromosomes) among different species: human, mouse, chicken, duck, and turkey (left side). The diploid chromosome numbers (2*n*) are denoted. m6A-related genes are indicated in the upper panel.

To correctly assess patterns of comparative collinearity on the chromosomal scale of different m6A-related orthologs, we selected a closely related species such as human and mouse (i.e., diverged about 40–80 million years from a common ancestor; Frazer et al., 2003), and compared it with some avian species including chicken, duck, and turkey that represent an evolutionary intermediate-level species (i.e., diverged about 310 million years; International Chicken Genome Sequencing Consortium, 2004). The m6A-related orthologs distribution revealed that they were allocated in various chromosomes (mainly autosomal in human) that were designated by their chromosome number (Figure 1). However, m6A-related orthologs distributed in both autosomal and the common sex chromosome in both sexes: chromosome X in mouse and chromosome Z in avian species (Figure 1).

*Vis-à-vis* human, the relative order of some orthologs (YTHDF3 and METTL3) was maintained in mouse chromosomes 3 and 14, respectively whereas some orthologs shared the same chromosome number with chicken such as YTHDF1, YTHDC1, and METTL14 that located at chromosomes 20, 4, and 4, respectively, (Figure 1). However, the loss of conserved synteny in the rest of m6A-related orthologs was clearly noticed. Loss of synteny was also observed among avian species with a minor conservation (certain conserved syntenies are present). Two m6A genes including METTL3 and METTL14 in turkey and METTL3 in duck remained uncharacterized and were absent in the current version of the Ensemble database.

### The Evolutionary Changes in Avian m6A Methyltransferase

**FIGURE 2:**
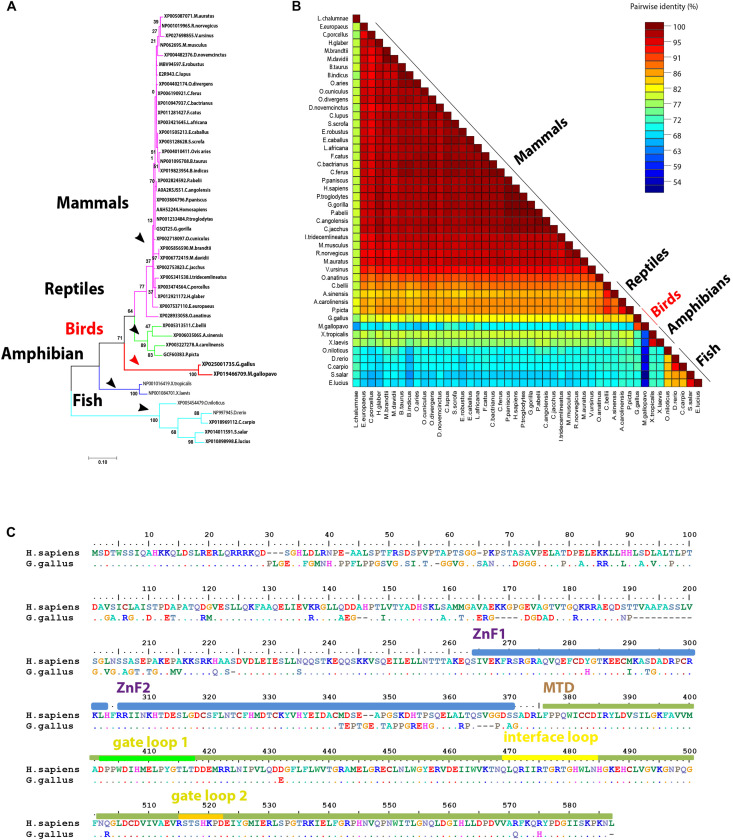
Structural comparison between human and some avian m6A writers. **(A)** Phylogenetic analysis of methyltransferase like-3 (METTL3) protein between various orthologs. The species were grouped by their orders and indicated by different colors. The phylogenetic tree was generated using MrBayes (http://mrbayes.sourceforge.net/). The name of the species and the accession of each protein are indicated. The avian proteins were marked by red branches. Bootstrap probabilities are denoted at the branch nodes. The scale bar at the bottom indicates the error rate. **(B)** Pairwise identity% plot between METTL3 protein and various orthologs was performed using an SDT program; the identity percentage was represented on the right side scale of the plot. **(C)** Sequence alignment of the entire METTL3 protein. The alignment was generated using Clustal W algorithm of MegAlign program (Lasergene, version 3.18). The species of comparison was indicated on the left side. Identical residues are indicated by dots, and sequence variation is denoted by a single-letter code. Zinc finger (ZnF) domains are highlighted by light blue bars. Methyltransferase domain (MTD) was highlighted by a dark green bar. MTD loops: gate loop 1, gate loop 2, interface loop are highlighted by green, yellow, and orange bars, respectively.

The main enzyme that catalyzes m6A modifications (methylation) in the majority of the mammalian species is METTL3 (Śledź and Jinek, 2016; Huang and Yin, 2018) with the help of another pseudo methyltransferase (METTL14; Wang et al., 2017) as well as WTAP (Schöller et al., 2018). The overall structure of the methyltransferase domain (MTD) resembles a butterfly. The methyltransferase complex is not well defined in the avian genome databases (NCBI, Ensemble) and instead a truncated version is identified in turkey (truncated protein). Unlike other m6A-related proteins, METTL3 was also not identified in wild birds. Phylogenetically, avian METTL3 clustered in a distinct group, which was separated from mammals and reptile’s clade on one side, and amphibians and fish on another side (Figure 2A). Regarding the amino acid homology percent of m6A writers, METTL3 witnessed the lowest percent identity (about 82%) in chicken (Figure 2B). Whereas phylogenetically, the remaining writers-complex showed the same pattern as METTL3, with higher homology over 90% in METTL14 and WTAP (Supplementary Figures S1, S2).

**FIGURE 3:**
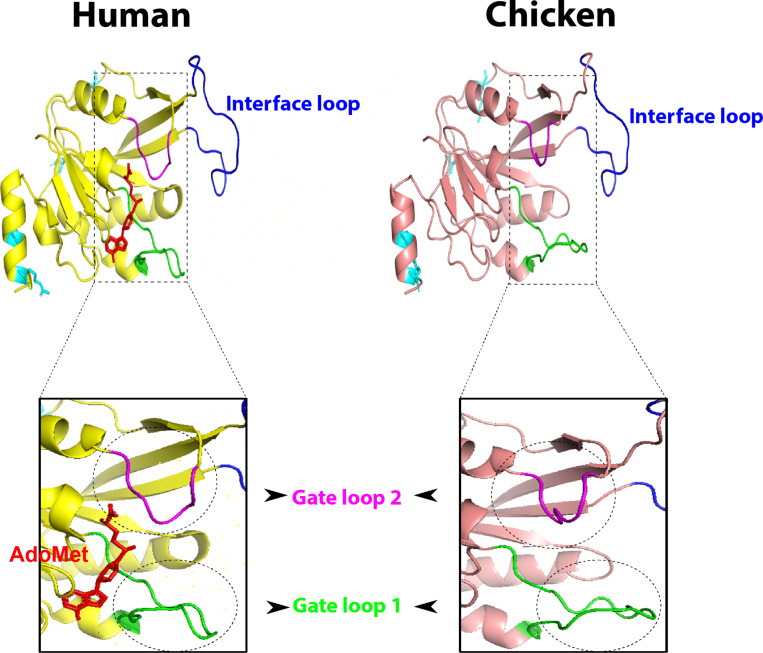
Overall three-dimensional (3D) structure comparison between human and chicken methyltransferase domains (MTDs) of methyltransferase like-3 (METTL3). MTDs of human METTL3 (PDB ID: 5IL1; left) harboring AdoMet (red) and the predicted chicken domain (right). The four recorded mutations are indicated by cyan sticks in both domains. MTD loops: gate loop 1, gate loop 2, interface loop are highlighted by green, blue, and magenta loops, respectively. The gate loops 1 and 2 are highlighted with black dashed borders.

Compared to human, chicken METTL3 revealed no detectable amino acid changes in the main loops (gate loop 1, gate loop 2, and interface loop; Figure 3). These main loops are responsible for functionality of m6A writers; however, several point mutations were encountered all over the protein domains such as in ZnF1, ZnF2, and MTDs. Interestingly, some loops revealed misfolding (Figures 2C, 3). Multiple sequence amino acid alignment of METTL14 revealed point mutations in MTD but did not affect the conserved vestigial catalytic EPPL motif. Likewise, WTAP protein acquired several mutations in the non-coiled coil domain (Supplementary Figure S3).

### Avian m6A Demethylases Reveal Distinct Phylogenetic Clustering From Mammalian Orthologs With Maintaining Overall Protein Structure Through Synonymous Mutations

**FIGURE 4:**
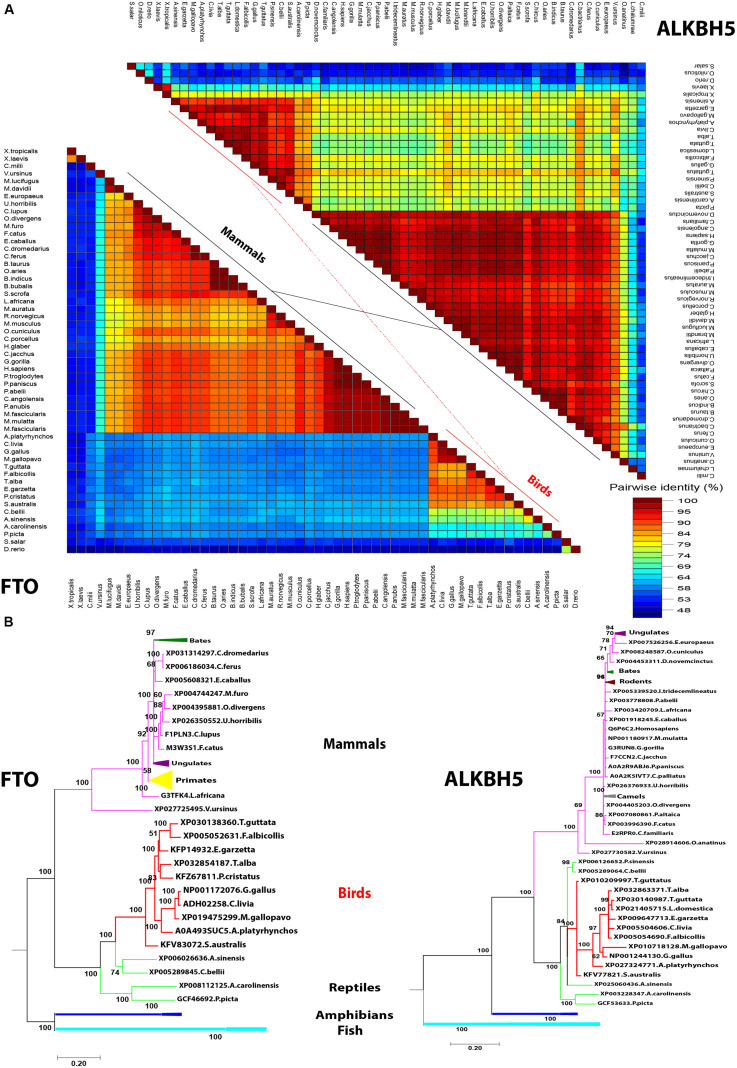
Pairwise distance and phylogenetic analysis of m6A demethylases’ orthologs. **(A)** Pairwise identity% plot between the erasers performed using an SDT program in different species; the percent identity was represented on the right side scale of the plot. **(B)** Phylogenetic analysis of m6A erasers in different species. The species were grouped by their orders and indicated by different colors. The phylogenetic trees were generated using MrBayes (http://mrbayes.sourceforge.net/). The name of the species and the accession of each protein are indicated. The avian proteins are marked by red branches. Bootstrap probabilities are denoted at the branch nodes. The scale bar at the bottom indicates the error rate.

In a trial to investigate the genotypic clustering of m6A demethylases in the investigated avian species, a collection of wide arrays of mammalian genes from a range of orders was included in the analysis: mammals, reptiles, amphibians, and fish. Upon comparing amino acid identity percentage among avian species compared with human counterparts, the homology among m6A interacting proteins in avian FTO was the lowest (55–64%), while the homology between human, and avian ALKBH5 was 74–82% (Figure 4A). Notably, fish m6A demethylases (ALKBH5 and FTO) shared 50% similarity with *Homo sapiens*. The Bayesian inference analysis classified m6A-erasers into clear avian and mammalian clusters. Intriguingly, the duck-genes showed higher divergence compared to chicken and turkey. The FTO and ALKBH5 of birds were grouped with reptiles; the former enzyme has a distinct clade, while the latter was interspersed with reptiles’ clade. Amphibians and fish were widely separated in both phylogenetic trees (Figure 4B).

**FIGURE 5:**
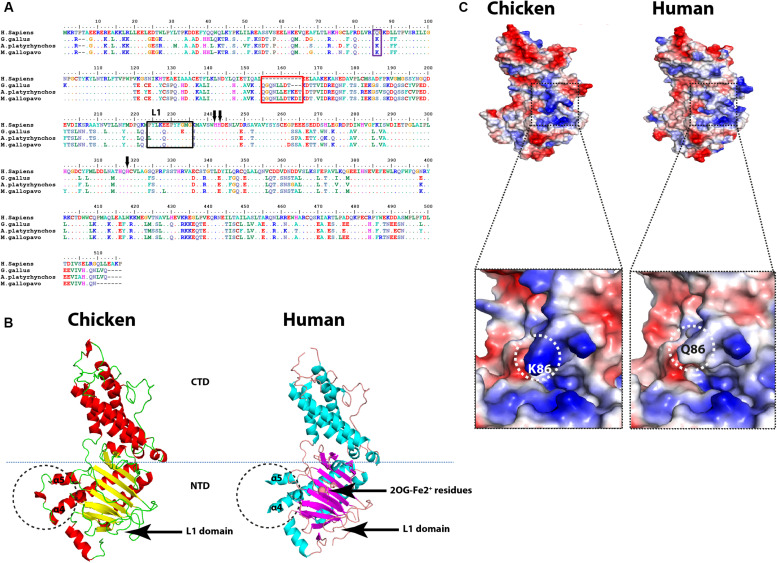
Structural comparison between human and avian fat mass and obesity-associated (FTO) proteins. **(A)** Sequence alignment of the entire FTOs. The alignment was generated using Clustal W algorithm of MegAlign program (Lasergene, version 3.18). The species of comparison are indicated on the left side. Identical residues are indicated by dots, and sequence variation is denoted by a single-letter code. The insertion is indicated by a red rectangle. The unique FTO Loop 1 (L1) is indicated by a black rectangle. Q86K mutation is labeled by a blue rectangle. The conserved (HxD.H) motif is indicated by black arrows. **(B)** Overall structure comparison between human FTO (PDB ID: 3LFM) and the predicted chicken FTO. The insertion is indicated by black dashed circles between α4 and α5 helices. CTD, *C*-terminal domain; NTD, *N*-terminal domain (active domain); L1, Loop 1 domain; 2OG-Fe^2+^ residues are indicated. **(C)** Electrostatic surfaces of the human FTO (PDB ID: 3LFM) and the predicted chicken FTO, enlarged detailed Q86K mutation is highlighted with black dashed borders.

Multiple amino acids’ sequence alignment of FTO from chicken, duck, turkey, and human revealed conservancy of Fe^2+^-coordinated residues (HxD.H; Figure 5A). Albeit avian species of interest possessed some consistent and unique amino acid mutations; however, the critical amino acid residues inside the catalytic pocket were located in the vicinity with sugar and purine ring of the m6A. Collectively, isoleucine 85, tyrosine 108, leucine 109, valine 228, serine 229, and tryptophan 230 showed no change in avian species (Figure 5A) that might reflect the same demethylation activity of FTO in avian species compared to *Homo sapiens*. Notably, putative analysis of the avian FTO proteins revealed E200Q mutation in the unique loop (L1) site which is characteristic to FTO among the ALKBH family proteins. Furthermore, the residue Q86K was observed in avian species. This notable alteration was found to enhance the binding affinity in human FTO mutant harboring this induced mutation (Zhang et al., 2019; Figures 5A,C). Moreover, in the exposed surface of FTO between α4- and α5-helices, unique 8, 11, and 11 amino acid inserts were observed in chicken, duck, and turkey, respectively (Figures 5A,B). Future studies are warranted to functionally annotate these substitutions in the avian m6A-associated genes.

Regarding ALKBH5, the iron-coordinated residues (HxD.H) residues were conserved in all analyzed species in this study; in contrast, out of all tested species, avian ALKBH5 recorded 19 amino acid residue deletions in the N-terminus in the non-catalytic domain (data not shown). The predicted three-dimensional (3D) structures of avian erasers revealed overall similar structures compared with reference known crystalized *Homo sapiens* structures. Taken together, avian m6A erasers shared the lowest sequence identity (about 55%) with maintaining its 3D structure among different mammalian species through synonymous structural mutations.

### Evolutionary Changes in Avian m6A Readers

**FIGURE 6:**
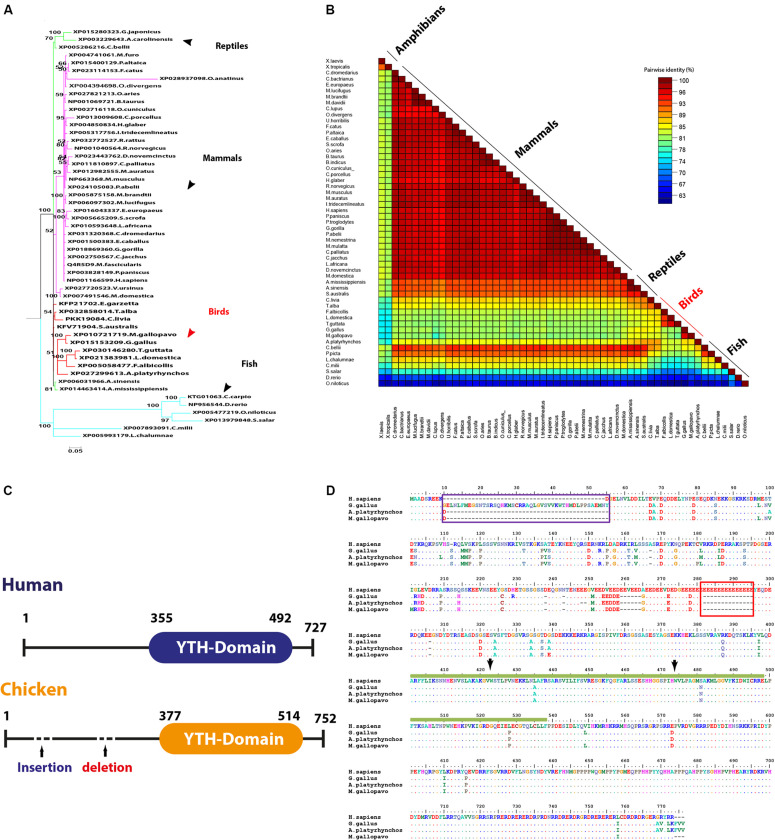
Structural comparison between human and some avian m6A readers. **(A)** Phylogenetic analysis of YTHDF2 protein between various orthologs. The species were grouped by their orders and indicated by different colors. The phylogenetic trees were generated using MrBayes (http://mrbayes.sourceforge.net/). The name of the species and the accession of each protein are indicated. The avian proteins are marked by red branches. Bootstrap probabilities are denoted at the branch nodes. The scale bar at the bottom indicates the error rate. **(B)** Pairwise identity% plot between YTHDC2 protein and various orthologs performed using an SDT program; the percent identity was represented on the right side scale of the plot. **(C)** Domain architectures of the YTHDC1 in *Homo sapiens* and chicken. **(D)** Sequence alignment of the entire of YTHDC1 proteins. The alignment was generated using Clustal W algorithm of MegAlign program (LaserGene, version 3.18). The species of comparison was indicated on the left side. Identical residues are indicated by dots, and sequence variation is denoted by a single-letter code. The insertion was indicated by a dark blue rectangle. The deletion was indicated by a dark red rectangle. YTH-domain was highlighted by amino acid under the dark green bar. The conserved aromatic cage residues are indicated by black arrows.

Five proteins that represent the well-documented YTH family in all species in the current study for analysis include YTHDF1, YTHDF2, YTHDF3, YTHDC1, and YTHDC2. Phylogenetic clustering witnessed the same grouping pattern as was observed with avian m6A writers that clustered in a separate and distinct group (Supplementary Figures S6, S7) except YTHDF2 (Figure 6A). Phylogenetically, the YTHDF2 was grouped with mammalian proteins and reptiles in the same clade; the other clade harbored fish. Interestingly, among all YTH-domain family, amphibians lack the YTHDF2 (Figure 6A). The homology percent between YTH family protein varied where the highest identity between human and chicken was observed between YTHDF3 (96%), followed by YTHDF2 (92%). Among YTH-containing proteins, the lowest percentage identity of avian proteins compared to *Homo sapiens* was noticed in YTHDC2 with 80–85% (Figure 6B); all percentages of identity for all the analyzed species in this study were presented separately in Supplementary Figures S4, S5.

Multiple amino acid sequence alignment revealed a number of consistent point mutations that reflect on the avian species evolutionary pattern allocated in all YTH proteins, which are documented as m6A-binding protein through their conserved YTH domain. No detectable mutation was recorded in YTHDF2 among avian species with *Homo sapiens*. A single E502D mutation was recorded in YTHDF3, and three consistent mutations were recorded in avian YTH-domain of YTHDF1 (Supplementary Figures S8, S9).

**FIGURE 7:**
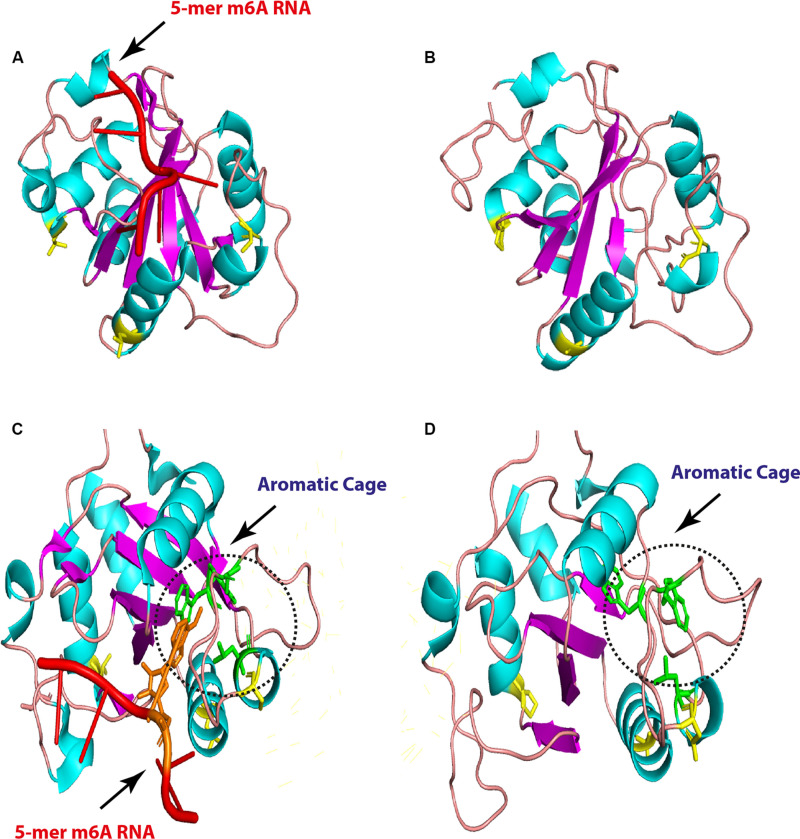
Overall three-dimensional (3D) structure comparison between human and chicken YTH-domain of YTHC1. YTH-domain of human YTHDC1 (PDB ID: 4R3I) harboring 5-mer m6A RNA (red; right) and the predicted chicken domain (left). The point mutations are indicated by yellow sticks in both domains **(A,B)**. An enlarged comparison between YTH-domain of human YTHDC1 (PDB ID: 4R3I) harboring 5-mer m6A RNA (red, the methylated adenosine labeled with orange), and the predicted chicken domain. The aromatic cages are indicated by green sticks in both domains and highlighted by black dashed circles. Point mutations are colored by yellow sticks in both domains.

Regarding YTHDC1, three critical hydrophobic residues were identified in deep cleft, tryptophan 377, 428, and leucine 439, at which m6A residue was left buried and found to be conserved in all analyzed species. Additionally, asparagine 363, 367, and serine 378 residues form hydrogen bonds with m6A nucleobase and were maintained in avian species, suggesting a conserved m6A recognition mode in majority of eukaryotes (Figure 6D). Remarkably, unique long insertion of 45 amino acid residues was detected in chicken YTHDC1. Furthermore, short 14 (glutamic acid, E) residue deletion was observed in avian species, along with singlet deletions in avian YTHDC1 compared to human proteins (Figure 6D). However, no publicly available crystal structure of the entire molecule of this reader was found to delineate their functions. Throughout the YTH domain of YTHDC1 protein, three consistent mutations were noticed in avian species (Figure 6D). The putative 3D structure witnessed a higher degree of similarity to crystalized human YTH-domain counterpart harboring m6A motif (Figure 7). However, a wider aromatic cage was detected in chicken YTH-domain. Three aforementioned mutations did not affect the predicted structure.

### The m6A-Associated Genes Are Virus-Inducible in Chicken

**FIGURE 8:**
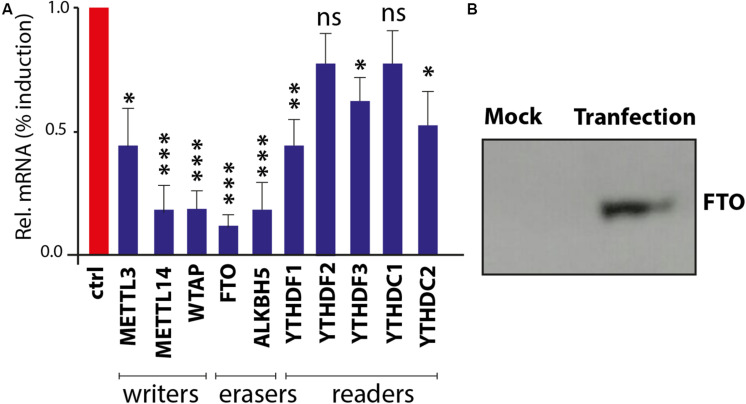
Confirmation and expression of m6A-associated genes in chicken fibroblasts. **(A)** Realtime PCR-based quantitative profile of all known m6A-writers, erasers, and readers. **(B)** Chicken fat mass and obesity-associated (FTO) gene expression in DF1 cells using Western blot.

It has been validated that the m6A-associated signatures are posttranscriptionally added on to the mRNA by “writers” and removed by “erasers” within the nucleus, whereas these epigenetic motifs are interpreted by “readers” in the cytoplasm. Intriguingly, influenza virus is one of the few RNA viruses that replicates in the nucleus and appears to acquire m6A marks on its genome (Courtney et al., 2017). In order to understand that *in silico* analyzed m6A genes are expressed and respond to viral infection, chicken fibroblasts were infected with avian influenza virus H9N2 strain UDL/08 and the expression profiles for the m6A-associated genes were determined. Transcriptomic analysis of the extracted RNA showed that influenza virus reduced the expression levels of m6A writers and erasers (Figure 8A). However, a relatively lower level of modulation was observed with m6A-readers. Additionally, western blot analysis of the FTO eraser indicated a robust expression (Figure 8B). These data indicate the existence and stimulus-induced regulation of m6A-associated genes in chicken, and the transcriptional suppression was least prominent in m6A-readers, indicating their important roles in viral gene expression. This first-level finding provides foundational information to underpin future functional annotations of the m6A-associated machinery in different avian species.

## Discussion

The ever-rising list of complete genomes from animals including chicken, duck, and turkey can be exploited in comparative genomics to unravel the evolutionary changes as chromosomal rearrangement and evolutionary rates with other species. Interestingly, chicken are considered an evolutionary intermediate-level species between the two extremes human and higher order mammals such as mouse, rat, cow (i.e., closely related), and fish as distantly related species (Furlong, 2005). Thus, chicken is the bridging animal model to fill the evolutionary gap between the two extremes. Furthermore, chicken is one of the chief animal models in seminal microbiological and immunological studies among 10,000 avian species (Vainio and Imhof, 1995; Jacobsen et al., 2012; Stewart et al., 2013). Answering questions related to chicken genome will fill gaps in the basic sciences and probably in agricultural fields. Therefore, we aim to provide comprehensive structural and genomic analyses for avian m6A machinery generally and chicken specifically as a preliminary step driving upcoming epigenetic researches in veterinary fields.

Chicken genome has approximately two fifths the genome size of human (International Chicken Genome Sequencing Consortium, 2004). Mapping genes to chromosomes in other animals might discover syntenic loci, where the genes of interest are located in a similar order along the chromosomes of different species. Complete genomes of chicken and human aligned segments tend to group in long blocks of conserved synteny (International Chicken Genome Sequencing Consortium, 2004). However, majority (7/10) of m6A-related orthologs lose their synteny in chicken compared to human. Although only 10 genes are very small for generalization in the whole chicken genome (about 23,000 genes), it might reflect insights to the evolutionary events that have shaped the chromosomes to these core genes. It was notable that some m6A-related orthologs were found syntenic, YTHDF2 protein exhibited phylogram with no out-grouped Aves cluster as was observed in other m6A proteins. This reflects a divergent evolution from a common ancestor and has a function not related to RNA metabolism. The lesser degree of conserved synteny between avian species themselves might be due to the difference in the diploid chromosome number from (2*n* = 39 to 40). However, deep functional and structure studies warrant further investigations.

Methylation of adenosines at the m6A site is controlled by MTDs of METTL3 and METTL14 cooperatively. Conservation of catalytic motif DPPW in gate loop 1 between human and chicken suggests the same mechanism of methyl transfer process from methyl moiety of AdoMet (methyl donor) to N6-position of the adenine residue of RNA between both species (Wang et al., 2016a, b). Even though mutations in the active sites and loops in METTL3 were not observed in chicken, however, the predicted 3D structures in gate loops 1 and 2 were found to be misfolded; no change was encountered in the interface loop. Mutations in ZnF domains (mainly ZnF2) were recorded in chicken; however, structural crystallographic studies are needed to investigate the accuracy of the prediction and the effect on methyltransferase activity (in METTL3 pocket for the AdoMet) in chicken compared to human to support the notions.

The METTL14 maintained the degenerate active site which plays non-catalytic roles through maintaining the integrity of the complex, as chicken shared the EPPL motif (equivalent to DPPW of METTL3) in the human counterpart that lacks the aromatic moiety required for interaction with the adenine for methylation (Śledź and Jinek, 2016; Wang et al., 2017). Likewise, WTAP protein witnessed also mutations that did not affect the main coiled-coil structure (*N*-terminal) and reflect the conserved function all among the species. However, the *C*-terminal was heavily mutated and thus requiring further investigations as there is no defined crystallographic structure generated to underpin their detailed structure and function. Unlike chicken, the unidentified METTL3 protein in majority of avian species may highlight the mechanisms of m6A addition. This is even more important given the role of METTL3 and METTL14 in optimal methyltransferase actions. However, future research is required to underpin this mechanim.

RNA demethylases belong to ALKB protein family of non-heme ferrous alpha ketoglutaric-dependent dioxygenases that are involved in various nucleic acid metabolic pathways including hydroxylation (Martinez and Hausinger, 2015). Both enzymes, ALKBH5 and FTO, share similar structures and m6A substrate preferences with different reaction pathways (Fu et al., 2013); however, FTO provides superiority in demethylation activity against m6A and more physiological substrates than ALKBH5 (Wei et al., 2018; Zhang et al., 2019). One of the unique structural features distinguishing FTO is an extra loop L1 (residues 213–224) documented to cover a side of the conserved jelly-roll motif (Han et al., 2010). Although avian species maintain L1 loop, they harbor a mutation that did not reflect on predicted 3D structure, it was believed that this loop is responsible for the hindrance to double-stranded nucleic acid for substrate selection (Han et al., 2010).

Zhang et al. (2019) have used crystallographic studies to identify that a mutation at 86 amino acid position to lysine (K) in human wild-type FTO (FTO^*WT*^) might act as a pincer through enhancing the binding affinity (1.5 fold using fluorescence anisotropy) to their substrates. Interestingly, K86 in avian FTO^*WT*^ proposes higher binding affinity of chicken FTO with their substrates, while conservation of critical residues in the catalytic pocket of chicken FTO in vicinity to the m6A nucleobase proposes that the demethylation activity might remain constant compared to the human counterpart. Moreover, the unique insertion between α4- and α5-helices suggests structural stability to avian compared to human FTO. Further biochemical studies are needed to support the notion that these residues in avian contribute strongly to substrate sequence recognition, stabilization, and function. Maintaining the conservancy of (HxD.H) motif in all ALKB family suggests the core function of the enzymes for coordinating Fe^2+^ is not only in intermediate-level evolutionary species as chicken but to the extreme, distantly related evolutionary species as in amphibians and fish as well (data not shown).

Human genome contains at least five YTH domain proteins that act as m6A readers to regulate RNA posttranscriptional functions (m6A-binding proteins). The five YTH domains have a selective preference to the consensus m6A motif mainly in (–1) position (i.e., the nucleotide preceding the methylated adenosine), mainly based on the fittingness for the protein binding pocket for nucleotide at -1 position (Li et al., 2014; Xu et al., 2014b, 2015; Liao et al., 2018). Conservation of certain residues, leucine 380, methionine 438, valine 382, and asparagine 383, in YTHDC1 between in both human and avian species suggests that avian prefers G in (–1) position *via* base-specific hydrogen bonds, and the presence of A at this position might mediate stearic clash with valine 382 (Xu et al., 2014b; Liao et al., 2018).

Remarkably, YTH domains reveal similar identities between each other, human YTHDF2–YTH domain share about 90% similarities to that of YTHDF1 counterpart in sequence, with maintaining the critical residues (i.e., the aromatic cage for m6A recognition; Xu et al., 2015). Multiple amino acid sequence alignments for avian readers as expected from their identity percentage showed the highest degree of similarities with human among m6A protein machineries that signify their core function in the evolutionary process and might regulate functions other than RNA metabolism. This also clarifies that YTHDF2 failed to cluster to distinct avian species, which harbor no mutation in YTH-domain compared to human. Additionally, a number of consistent point amino acid mutations were recorded but did not affect the highly conserved aromatic cage at which m6A adenine moiety is sandwiched in other YTH-domain proteins; mutations at these cage were recorded to abolish the readers functions (Li et al., 2014).

Although the avian YTHDC1-YTH domain is similar to that of human and the observed three amino acids did not change the predicted 3D structures, the binding pocket for the favored recognition sequence of the GG (m6A)C (Xu et al., 2014b) was maintained in the aromatic cage. However, the cage carried slightly widened compared to human cage. The effect of the unique chicken 45 amino acid insertion and 14 amino acid deletions in all analyzed avian species compared to human cannot be assessed due to unavailability of complete YTHDC1 and YTHDC2. Further avian structural and binding studies might unveil a general function of the avian YTH domains.

Although virus-induced expression analysis confirmed the transcription of m6A-associated genes in chicken fibroblasts, future functional studies are needed in different avian species. Ultimately, detailed structural, functional, and regulatory roles of methylated adenosine m6A remain an enigma and depending on comparative genomic studies might uncover the difference between species. These include existing and important novel mechanistic insights into the roles of m6A in cell and virus life cycle. Detailed future studies on m6A modifications in avian species could provide valuable information for improvement of basic biomedical sciences and veterinary research fields.

## Materials and Methods

### Sequence Data Mining

Ten N^6^-methyladenosine (m6A)-related proteins were analyzed and compared in this study. Various species represent main orders of class Mammalia, including primates, rodents, ungulates, carnivores, chiroptera (bats), and insectivores. Species of interest from class Aves included domestic and wild and ornamental birds. In addition to representative species from reptiles, amphibians and various cartilaginous and bony fish were included in the evolutionary analyses. m6A-related proteins were denoted by amino acid sequences that were retrieved from the Uniprot^1^ and NCBI (National Center for Biotechnology Information, www.ncbi.nlm.nih.gov) databases in FASTA format.

### Evolutionary Distance, Multiple Alignment, and Phylogenetic Analysis for Cross-Species Comparisons

Multiple sequence alignments (MSAs) of the corresponding sequences of each amino acid of interest in different species were performed using Clustal W algorithm of the MegaAlign program, Lasergene Software, version 3.18 (DNAStar, Madison, WI, United States), for identification of mutation hot spots and prediction of amino acid changes. Time points of divergence and evolutionary rates between m6A-related genes in various orthologs were estimated using at least two different evolutionary algorithms: Bayesian Markov chain Monte Carlo (MCMC) inference (Drummond et al., 2012) and the Maximum likelihood (ML) analysis (Tamura et al., 2013). For Bayesian inference, MrBayes algorithm was adopted using the Jones-Taylor-Thornton (JTT) amino acid substitution model with a gamma distribution and invariant sites (JTT + Γ4 + I). Ten independent runs with the initial 25% burn-in were used. Each run consisted of two independent files with each 400,000 MCMC-sampled trees. The trees were compared with ML trees, which were performed using MEGA 6.0 software for prediction of the best evolutionary model. A total of 1,000 bootstrap replicates were used to ensure the robustness of the tree. Percent identity and divergence changes for cross-species comparison were generated by SDT (sequence demarcation tool, available from http://web.cbio.uct.ac.za/SDT) aligned with MUSCLE mode (Muhire et al., 2014).

### Molecular Location and Molecular Modeling

Ensemble genome browser^2^ was used to determine the relative genetic loci of 10 N6-methyladenosine (m6A) genes of interest in different avian and mammalian species. The m6A-related genes were annotated to the corresponding chromosome through region comparison in five species (orthologs): human, mouse, chicken, duck, and turkey. PSIPRED web server^3^ was used to predict the 2D structure and function. 3D structure prediction was performed using PHYRE2 web server (Yang et al., 2015) and confirmed with I-Tasser (Kelley and Sternberg, 2009) to predict each representative protein with the intensive mode. The PyMOL 1.3 (The PyMOL Molecular Graphics System, Version 1.3 Schrodinger, LLC) and Chimera^4^ were used to annotate and visualize the known crystalized *Homo sapiens* structures as well as predicted protein structures.

### Realtime PCR or Quantitative Expression of m6A Genes

Chicken embryo fibroblasts (DF-1) were infected with 1.0 multiplicity of infection (moi) of low pathogenic avian influenza virus H9N2 for 24 h. The total RNA was extracted using conventional TRIzol reagents (Invitrogen, Carlsbad, CA, United States). The NanoDrop 1000 (PEQLAB BioTechnologie GMBH, Erlangen, Germany) was used to assess the amount and quality of the TRIzol-extracted RNA. In order to assess the level of transcription of the m6A-associated genes, a total of 200 ng of RNA was used in SuperScript^®^ III Platinum^®^ SYBR^®^ Green One-Step qRT-PCR Kit (Invitrogen, Carlsbad, CA, United States). The fold change was estimated with comparative Ct values of the m6A-associated genes and 28S rRNA. The real-time PCR reaction was performed in the ABI 7500 light cycler and run on the profile: 50°C for 15 min followed by 95°C for 2 min. Thereafter, 40 cycles were run each for 95°C for 15 s and 60°C for 30 s. The melting curve was determined at 95°C for 15 s, 60°C for 1 min, 65°C for 10 s, and 95°C for 15 s.

### Western Blotting

The cDNA, fused with the FLAG tag, encoding chicken FTO was chemically synthesized and cloned into pCAAGs vector under CMV promoter as part of our ongoing m6A and influenza virus project. The pCAGGs-FTO plasmid or empty plasmid was transfected into DF-1 cells using Lipofectamine2000 (Invitrogen) for 48 h. After 48 h posttransfection, cells were washed with ice-cold phosphate buffered saline. Lysis for the transfected and control cells was performed by adding 100 μl lysis buffer; ice-cold Nonidet P-40 buffer completed with protease inhibitors cocktail for 15 min on ice. Then cells were scraped off to ensure complete disruption. Lysates were then left on ice for 10 min, followed by centrifugation at 13,000 rpm for 10 min at 4°C. The supernatants were denatured using Laemmli buffer [containing 0.05% β-mercaptoethanol (BME)] at 98°C for 5 min before loading onto a tris–glycine sodium dodecyl sulfate (SDS) 10% polyacrylamide gel. The separated proteins were transferred onto polyvinylidene difluoride membranes. After transfer, immunoblots were blocked with 5% skimmed milk and then incubated with primary monoclonal rabbit FLAG-tagged antibody (Abcam ab1162). Then, the blots were incubated with peroxidase-conjugated anti-rabbit secondary antibodies (Abcam ab6721) and visualized using chemiluminescence (Chemidoc, BioRad, Hercules, CA, United States).

## Data Availability Statement

Publicly available datasets were analyzed in this study. This data can be found here: www.uniprot.org; www.ncbi. nlm.nih.gov.

## Author Contributions

MM contributed to the conceptualization. MB, MR, and MM contributed to the formal analysis and investigation. MB contributed to writing the original draft. MM and MR contributed to writing, reviewing, and editing. MB and MM contributed to the resources. MM contributed to the supervision. All authors contributed to the article and approved the submitted version.

## Conflict of Interest

The authors declare that the research was conducted in the absence of any commercial or financial relationships that could be construed as a potential conflict of interest.

## Footnotes

1 www.uniprot.org

2 www.ensembl.org

3 http://bioinf.cs.ucl.ac.uk/psipred/

4 https://www.cgl.ucsf.edu/chimera/
