# Supplementary material for: Structural and Virus Regulatory Insights Into Avian N6-Methyladenosine (m6A) Machinery
Source: Front Cell Dev Biol. 2020 Jul 15;8:543. doi: 10.3389/fcell.2020.00543 (PMC7373739; doi:10.3389/fcell.2020.00543)
Supplement: Supplementary file 1 [file Presentation_1.PPTX]

## Slide 1
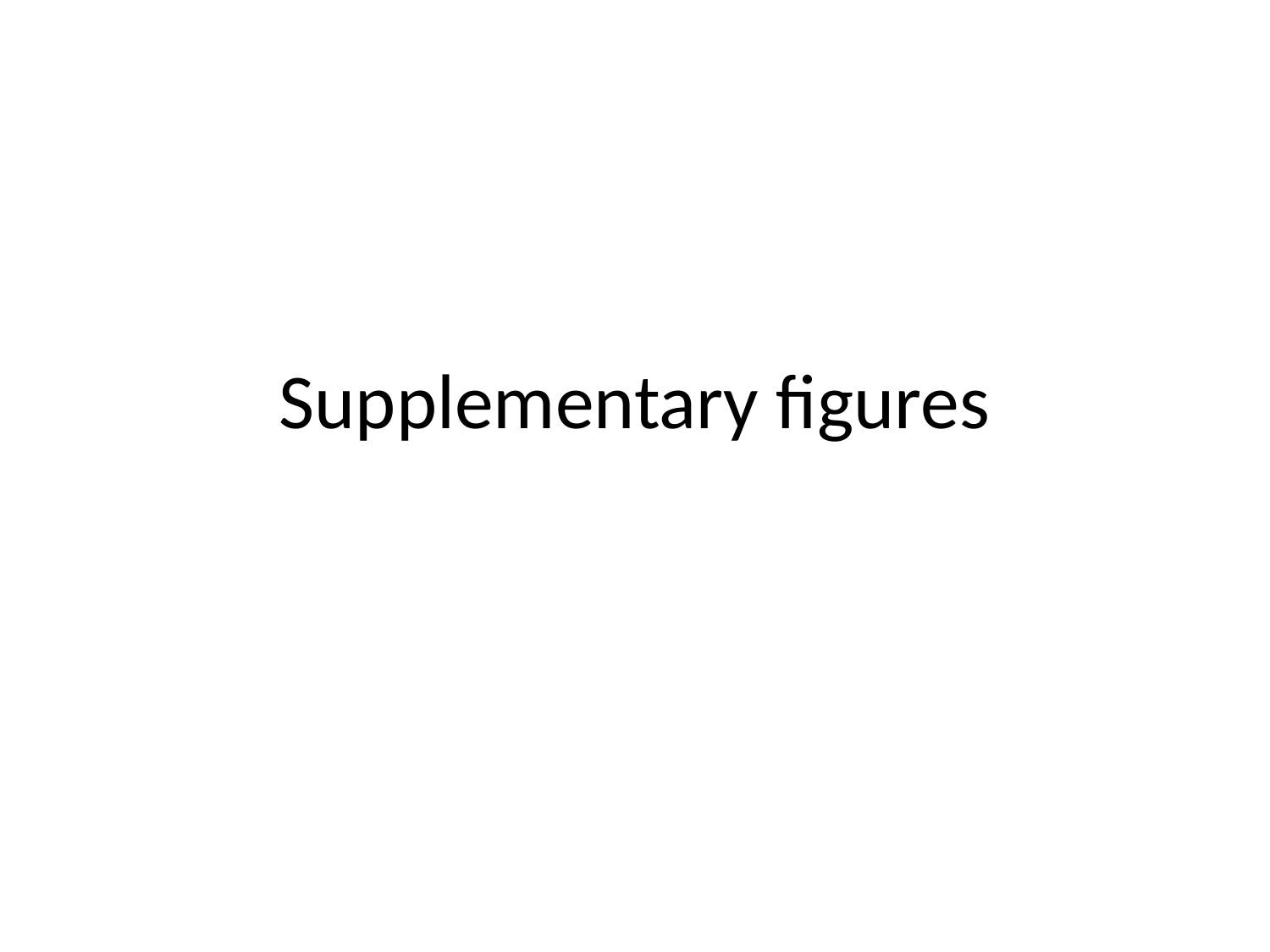

# Supplementary figures

## Slide 2
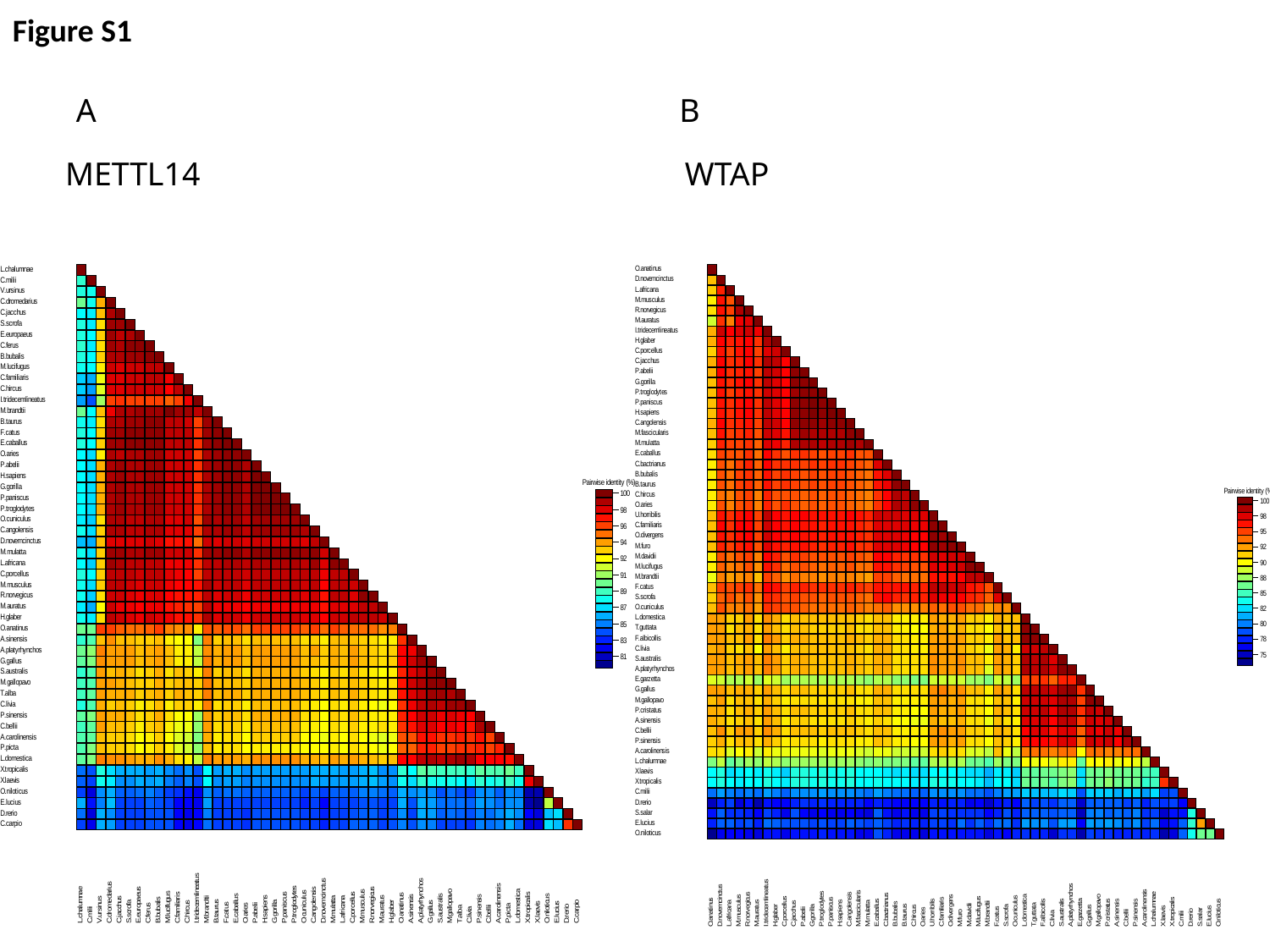

Figure S1
A
B
METTL14
WTAP

## Slide 3
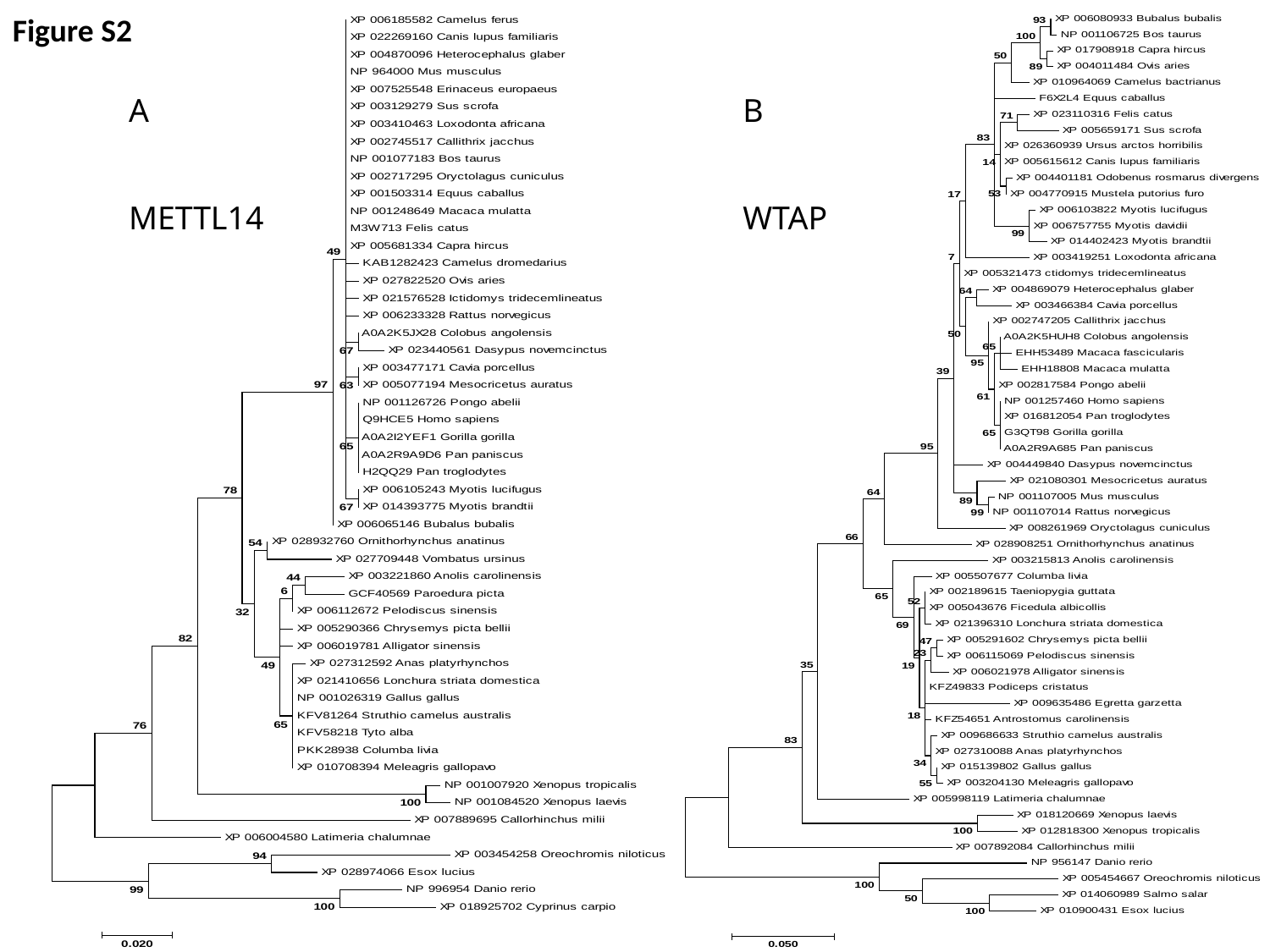

Figure S2
A
B
METTL14
WTAP

## Slide 4
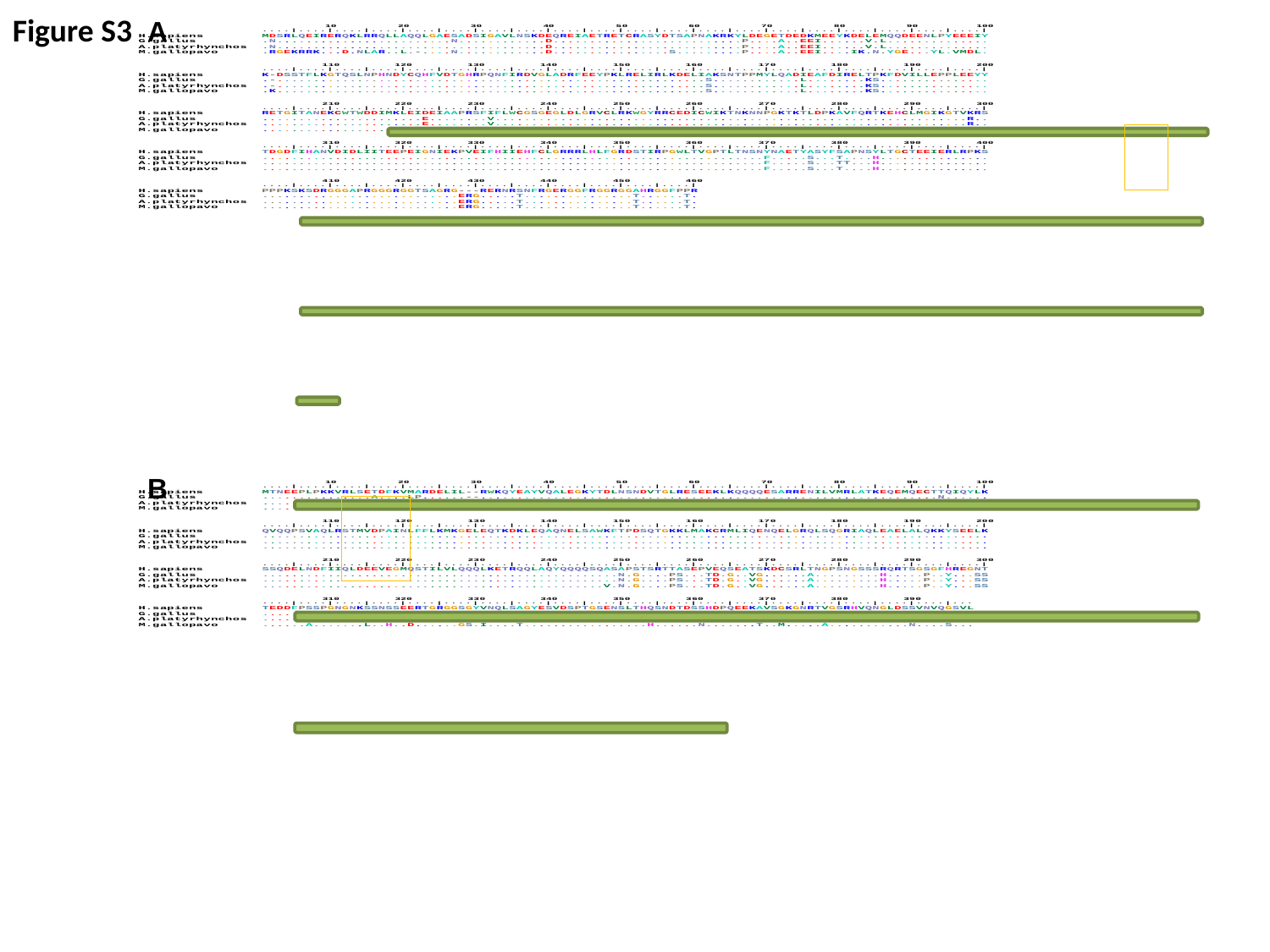

Figure S3
A
B

## Slide 5
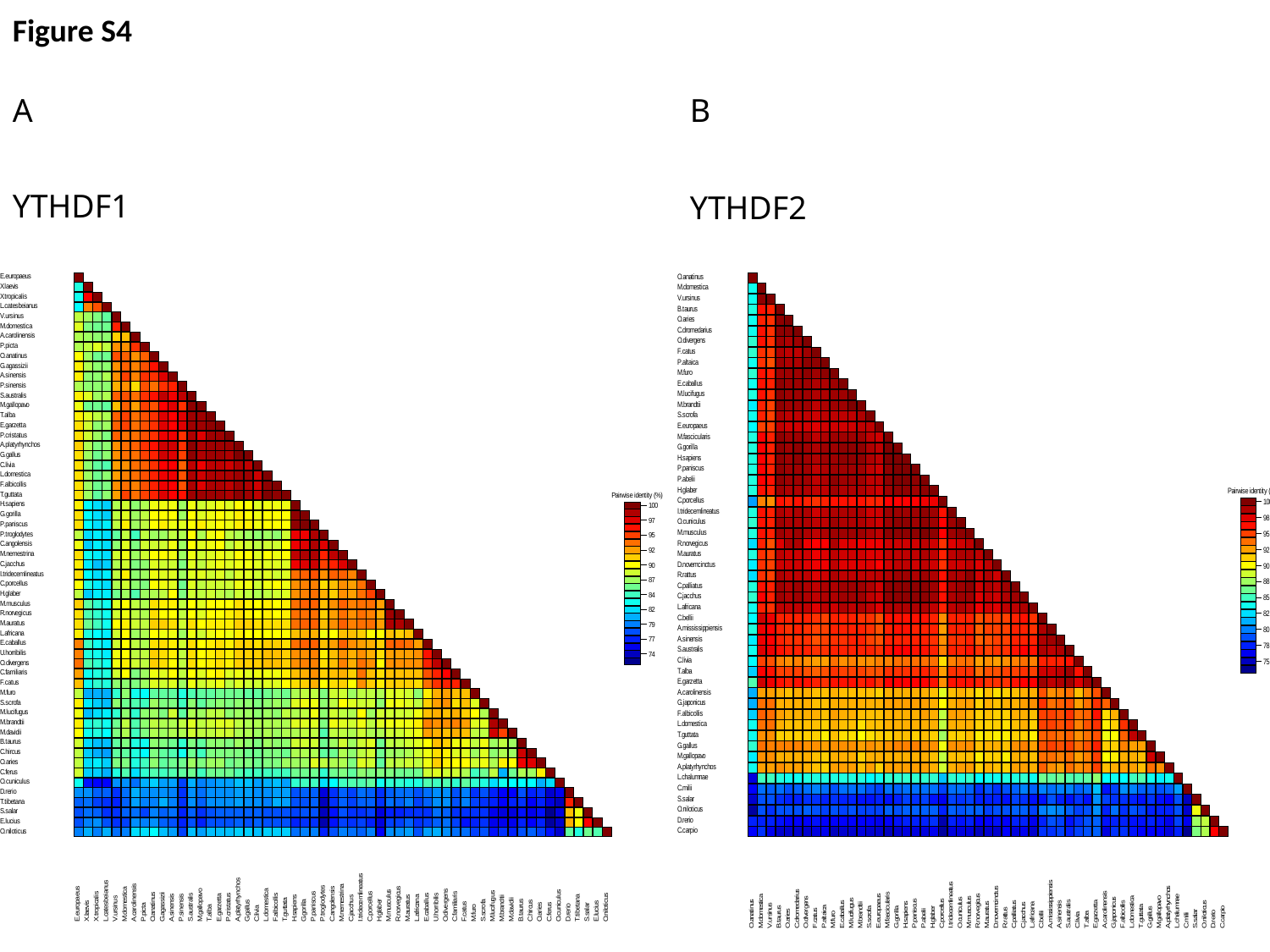

Figure S4
A
B
YTHDF1
YTHDF2

## Slide 6
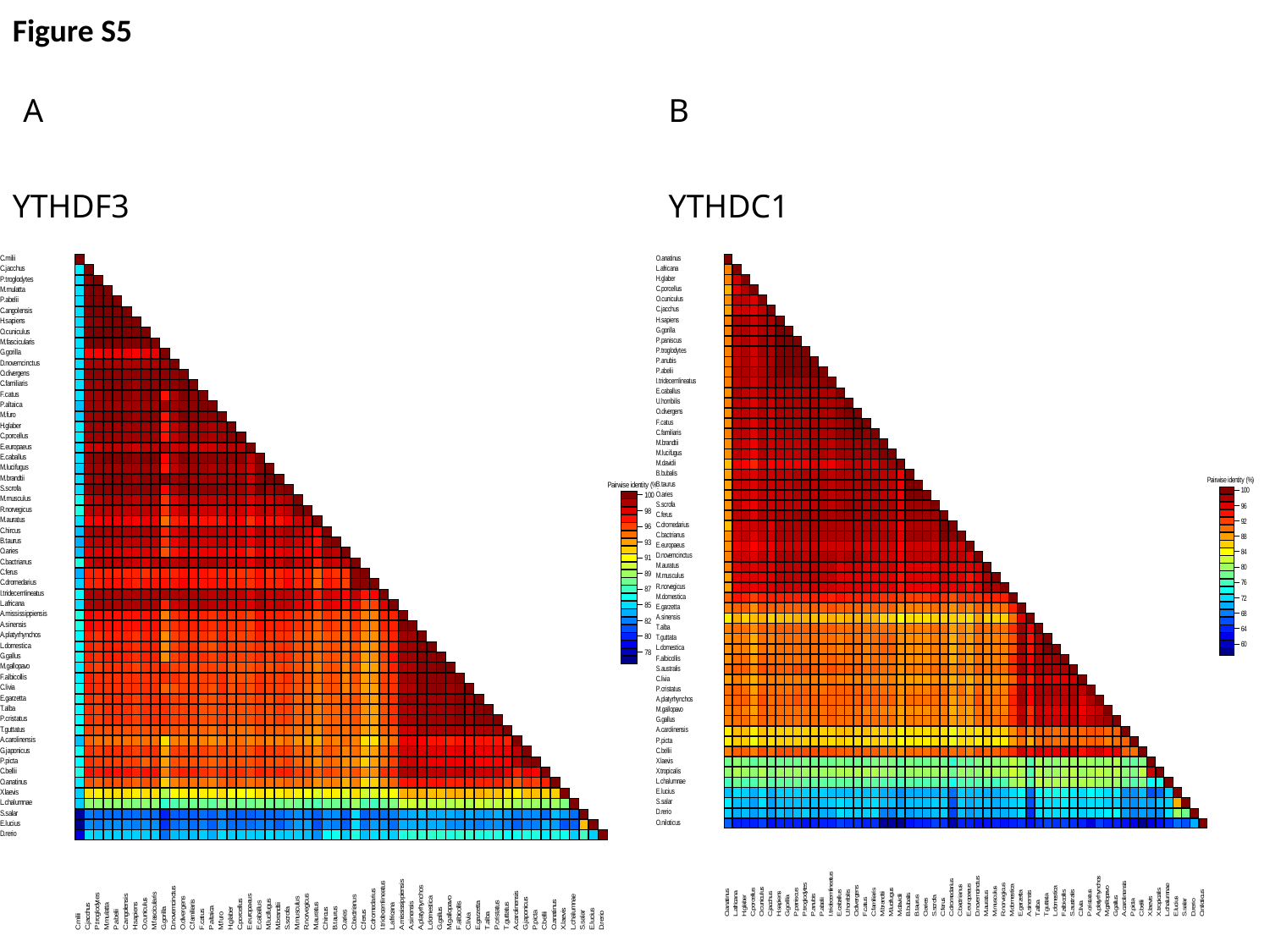

Figure S5
A
B
YTHDF3
YTHDC1

## Slide 7
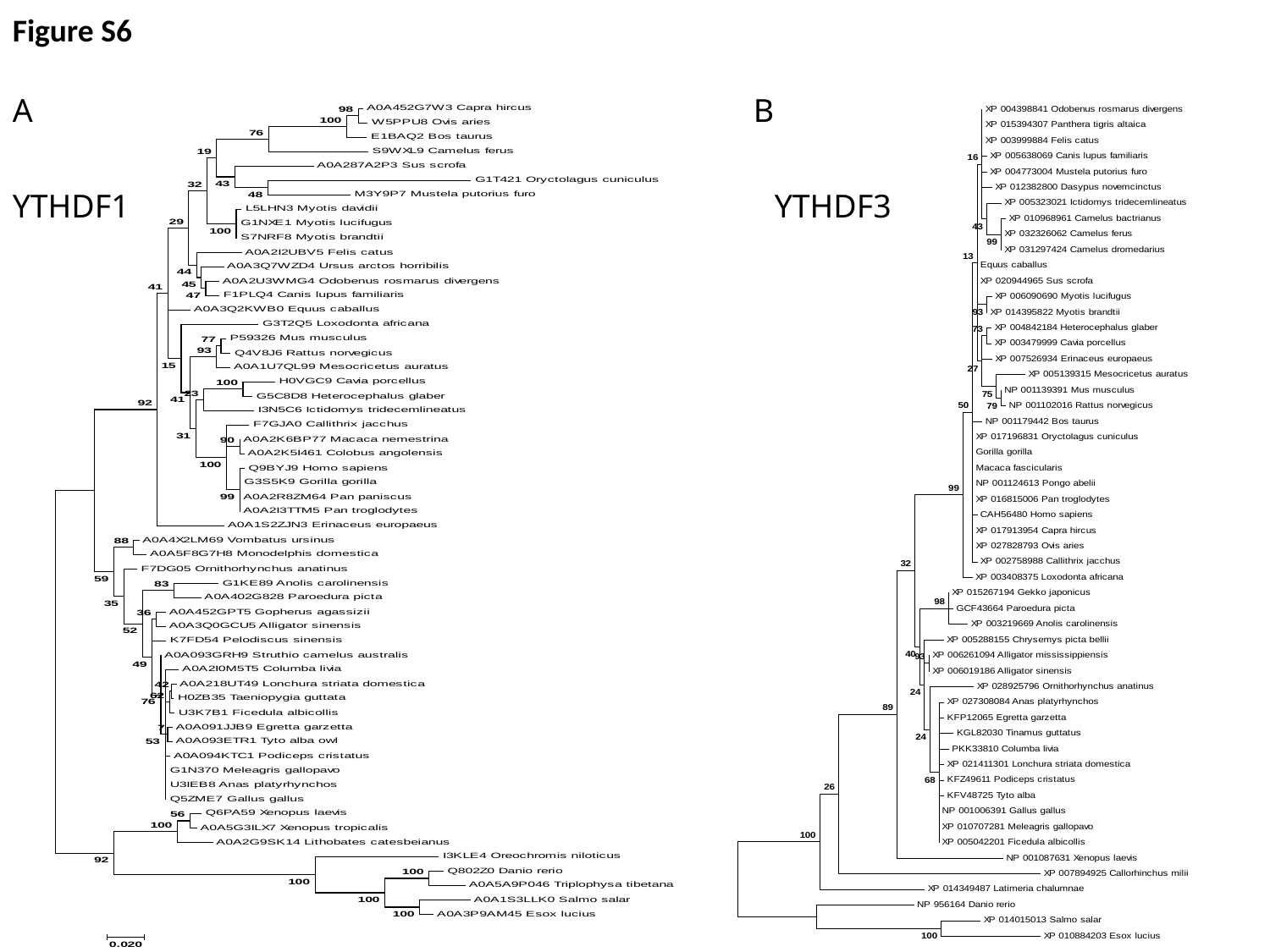

Figure S6
A
B
YTHDF1
YTHDF3

## Slide 8
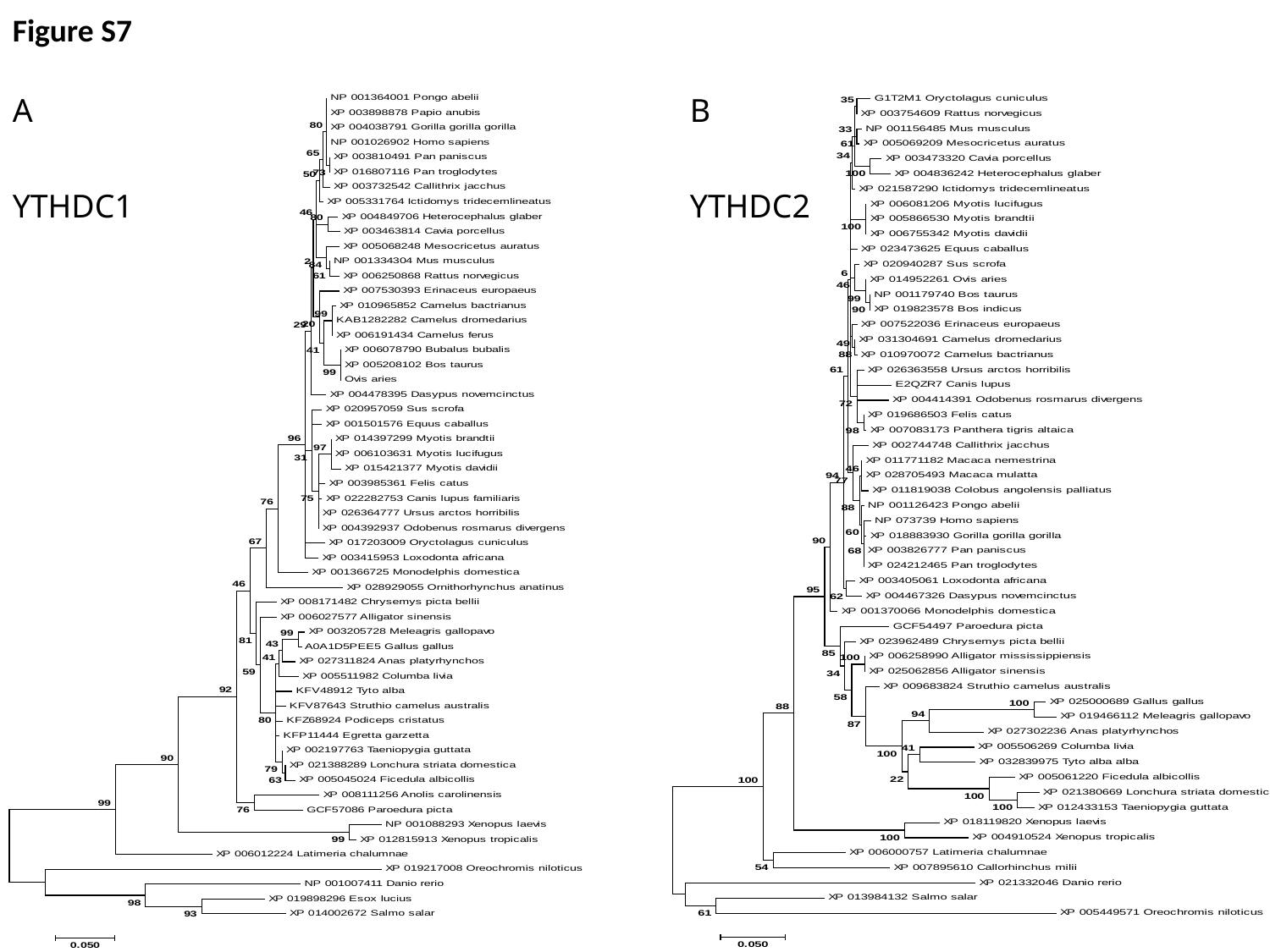

Figure S7
A
B
YTHDC1
YTHDC2

## Slide 9
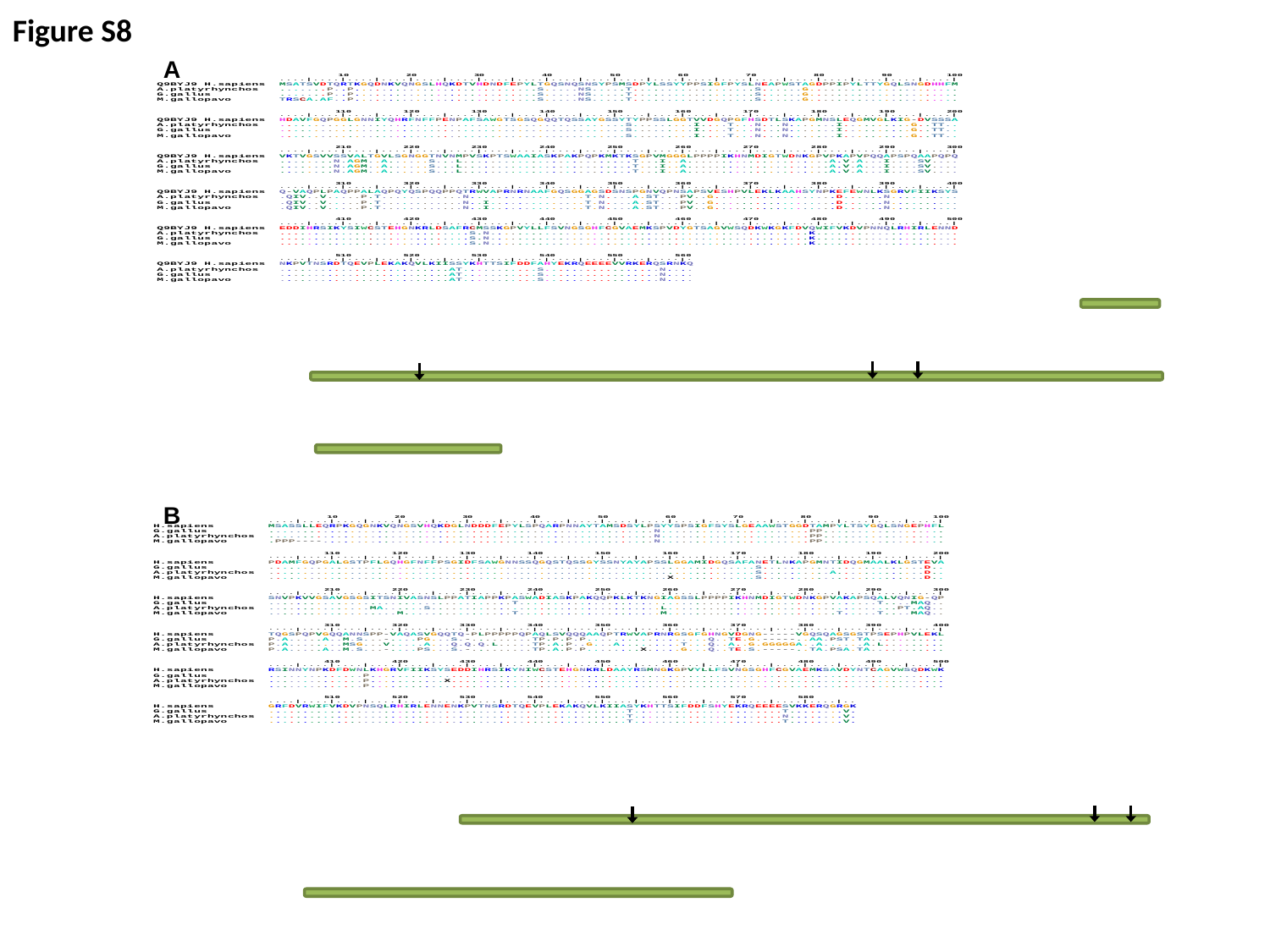

Figure S8

## Slide 10
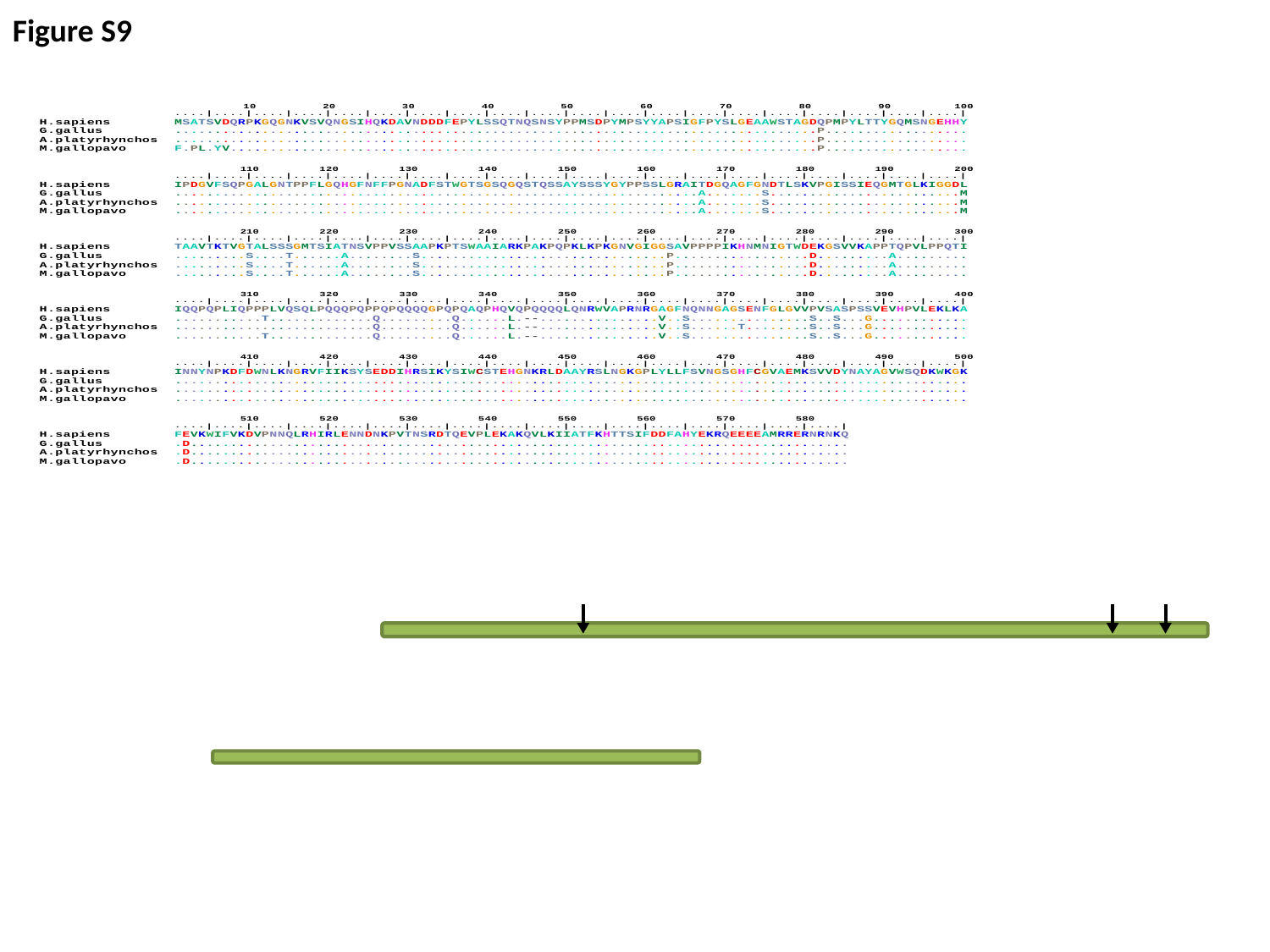

Figure S9
