# Supplementary material for: Structural and Virus Regulatory Insights Into Avian N6-Methyladenosine (m6A) Machinery
Source: Front Cell Dev Biol. 2020 Jul 15;8:543. doi: 10.3389/fcell.2020.00543 (PMC7373739; doi:10.3389/fcell.2020.00543)
Supplement: Supplementary file 2 [file Table_1.DOCX]

**Supplementary figure Notes:**

**Supplementary figure S1:** Pairwise identity of m6A writers between various orthologs. Pairwise identity % plot between m6A writers performed using a SDT program; A) METTL14, B) WTAP in different species, the percent identity represented on right side scale of the plot.

**Supplementary figure S2:** Phylogenetic comparison m6A writers between various orthologs. Phylogenetic analysis of m6A writers; A) METTL14, B) WTAP. The phylogenetic trees were generated using maximum-likelihood method in MEGA 6.0 program. The name of the species and the accession number of each protein are indicated. Bootstrap probabilities are denoted at the branch nodes. The scale bar at the bottom indicates the error rate.

**Supplementary figure S3:** Amino acid comparison between human and some avian m^6^A writers. Sequence alignment of the entire METTL14 (A), WTAP (B). The alignments were generated using Clustal W algorithm of MegAlign program (Lasergene, version 3.18). The species of comparison are indicated in the left side. Identical residues are indicated by dots and sequence variation is denoted by a single-letter code. MTD- domains (A), Coiled-coil (B) were highlighted by green bars. The conserved (EPPL) motif (A), nuclear localization signal (NLS) (B) are indicated by orange rectangles.

**Supplementary figure S4:** Pairwise identity of m6A readers between various orthologs. Pairwise identity % plot between m6A readers performed using a SDT program; A) YTHDF1, B) YTHDF2 in different species, the percent identity represented on right side scale of the plot.

**Supplementary figure S5:** Pairwise identity of m6A readers between various orthologs. Pairwise identity % plot between m6A readers performed using a SDT program; A) YTHDF3, B) YTHDC1 in different species, the percent identity represented on right side scale of the plot.

**Supplementary figure S6:** Phylogenetic comparison of m6A readers between various orthologs. Phylogenetic analysis of m6A readers; A) YTHDF1, B) YTHDF3. The phylogenetic trees were generated using maximum-likelihood method in MEGA 6.0 program. The name of the species and the accession number of each protein are indicated. Bootstrap probabilities are denoted at the branch nodes. The scale bar at the bottom indicates the error rate.

**Supplementary figure S7:** Phylogenetic comparison of m6A readers between various orthologs. Phylogenetic analysis of m6A readers; A) YTHDC1, B) YTHDC2. The phylogenetic trees were generated using maximum-likelihood method in MEGA 6.0 program. The name of the species and the accession number of each protein are indicated. Bootstrap probabilities are denoted at the branch nodes. The scale bar at the bottom indicates the error rate.

**Supplementary figure S8:** Sequence alignment of the entire of A) YTHDF1 B) YTHDF2 proteins. The alignment was generated using Clustal W algorithm of MegAlign program (Lasergene, version 3.18). The species of comparison are indicated in the left side. Identical residues are indicated by dots and sequence variation is denoted by a single-letter code. YTH- domains were highlighted by green bars. The conserved tryptophan (W) motifs are indicated by black arrows.

**Supplementary figure S9:** Sequence alignment of the entire of YTHDF3 proteins. The alignment was generated using Clustal W algorithm of MegAlign program (Lasergene, version 3.18). The species of comparison indicated in the left side. Identical residues are indicated by dots and sequence variation is denoted by a single-letter code. YTH- domains were highlighted by dark green bars. The conserved tryptophan (W) motifs are indicated by black arrows.

End
